# Association between Mitogen-Activated Protein Kinase Kinase Kinase 1 Polymorphisms and Breast Cancer Susceptibility: A Meta-Analysis of 20 Case-Control Studies

**DOI:** 10.1371/journal.pone.0090771

**Published:** 2014-03-04

**Authors:** Qiaoli Zheng, Jingjia Ye, Haijian Wu, Qing Yu, Jiang Cao

**Affiliations:** 1 Clinical Research Center, The Second Affiliated Hospital, Zhejiang University School of Medicine, Hangzhou, Zhejiang Province, China; 2 Department of Neurosurgery, The Second Affiliated Hospital, Zhejiang University School of Medicine, Hangzhou, Zhejiang Province, China; 3 Department of Surgical Oncology, The First Affiliated Hospital, Zhejiang University School of Medicine, Hangzhou, Zhejiang Province, China; Sanjay Gandhi Medical Institute, India

## Abstract

**Background:**

The genome-wide single-nucleotide polymorphisms (SNPs) profiles can be used as diagnostic markers for human cancers. The associations between mitogen-activated protein kinase kinase kinase 1 (*MAP3K1*) SNPs rs889312 A>C, rs16886165 T>G and breast cancer risk have been widely evaluated, but the results were inconsistent. To derive a conclusive assessment of the associations, we performed a meta-analysis by combining data from all eligible case-control studies up to date.

**Methods:**

By searching PubMed, ISI web of knowledge, Embase and Cochrane databases, we identified all eligible studies published before September 2013. Odds ratios (ORs) with 95% confidence intervals (CIs) were used to assess the strength of associations in fixed-effect or random-effect model. False-positive report probability (FPRP) was calculated to confirm the significance of the results.

**Results:**

A total of 59670 cases in 20 case-control studies were included in this meta-analysis. Significant associations with breast cancer risk were observed for SNPs rs889312 and rs16886165 polymorphisms with a per-allele OR of 1.11 (95% CI: 1.09–1.13) and 1.14 (95% CI: 1.09–1.20) respectively. For rs889312, in subgroup analysis by ethnicity, significant associations were identified in Europeans and Asians, but not in Africans. When stratified by estrogen receptor (ER) expression status, rs889312 was associated with both ER-positive and ER-negative breast cancers. Results from the FPRP analyses were consistent with and supportive to the above results.

**Conclusions:**

The present meta-analysis suggests that rs889312-C allele and rs16886165-G allele might be risk factors for breast cancer, especially in Europeans and Asians.

## Introduction

Globally breast cancer is the most commonly diagnosed cancer and the leading cause of cancer death in females, with an estimation of 1 million new cases and over 400,000 deaths per year [1]. Breast cancer is a genetically heterogeneous disease following a polygenic mode of inheritance. A better understanding of the genomic variations pertaining to the disease will eventually lead to improved diagnostic and treatment strategies for breast cancer patients. Evidence has been accumulated that mutations in several high and moderate penetrance genes, such as *BRCA1*, *BRCA2*, *ATM*, *CHEK2*, *BRIP1* and *PALB2*, contribute to breast cancer susceptibility. However, these mutations explain only a small portion of the disease risk and the majority of genetic variations that contribute to the breast cancer risk remains unclarified, especially those of low penetrance genes. Genome-Wide Association Study (GWAS), an examination of disease-related genetic variations across the human genome, provides a powerful tool in identifying such mutations/polymorphisms genome-wide. Some common low penetrance genetic variants that might confer increased risk to breast cancer have been identified recently [2], [3].

Mitogen-activated protein kinase kinase kinase 1 (*MAP3K1*) gene, which is located in the chromosome 5q11.2, encodes a serine/threonine kinase which is involved in the mitogen-activated protein kinase (MAPK) signaling pathway. MAPK signal transduction regulates the transcription of important cancer genes including *c-Myc*, *c-Elk1*, *c-Jun* and *c-Fos* [4], [5]. The single-nucleotide polymorphism (SNP) rs889312 in *MAP3K1* was identified to be associated with breast cancer risk by GWAS [5], with confirmation of the association in European ancestry population by another study [6]. A GWAS conducted in subjects of European descendant reported that the minor allele of *MAP3K1* rs16886165 was associated with increased risk of breast cancer under heterozygote codominant and homozygote codominant genetic models [7].

Up to date, a number of studies have investigated the relationship between polymorphisms in *MAP3K1* and breast cancer or its malignant phenotypes, whereas the results were inconsistent and inconclusive in populations of diverse ethnicities, especially among Asians and Africans [8], [9]. The current GWASs were mainly conducted in people of European ancestry, and most single studies with insufficient sample sizes were not powerful in detecting the minor effects of low penetrance alleles on breast cancer risk. Therefore, the meta-analysis presented here aims to summarize the available evidence for the genomic variants in *MAP3K1* and intends to provide the highest level of evidence for the association between breast cancer risk and *MAP3K1* (rs889312 and rs16886165) polymorphisms among diverse ancestry populations.

## Materials and Methods

### Literature search strategy

We conducted a comprehensive literature search in Pubmed, ISI web of knowledge, Embase and Cochrane databases up to September 2013 using the following terms: “breast cancer” or “breast carcinoma” and “*MAP3K1*” or “mitogen-activated protein kinase kinase kinase 1” or “rs889312” or “rs16886165” and “polymorphism” or “variation”. All potentially relevant studies were retrieved, and the abstracts were screened to exclude clearly irrelevant studies. The remaining articles were read to determine whether they contained information on the topic of interest. We also examined the reference lists from the main reports and reviews to identify additional relevant studies.

### Selection criteria

Eligible studies had to meet the following criteria: (1) original papers containing independent data which have been published in peer-reviewed journals, (2) case-control studies which evaluated the association between breast cancer risk and *MAP3K1* (rs889312 and rs16886165) polymorphisms, (3) had odds ratio (OR) with its 95% confidence interval (CI) or genotype distribution information for estimating OR (95% CI).

### Data extraction

Two investigators independently reviewed and extracted data with any discrepancies resolved by consensus. For each eligible study, the following data were extracted: first author's surname, year of publication, country of origin, population ethnicity, source of control subjects, genotyping method, age, the values of OR with its 95% CI, total numbers of cases and controls, the genotype counts in cases and controls, matching criteria, whether genotype distribution of control group was consistent with Hardy-Weinberg equilibrium (HWE). Ethnic groups were categorized as European, Asian and African (i.e. people of European, Asian and African ancestry). Different case-control groups in one study were considered as independent studies if the data was available.

### Quality assessment

Methodological quality was assessed using a classification method known as “extended-quality score”. The criteria cover diagnostic criteria, the degree of matching of controls, genotyping examination, Hardy-Weinberg equilibrium in the control population, and bias in data processing. Each paper was scored of ‘high’, ‘median’ or ‘poor’ quality. Detailed procedure of the quality assessment was performed according to literature [10].

### Statistical methods

The meta-analysis evaluated the association between rs889312 and the risk of breast cancer, for the: (1) allele contrast model (C vs. A), (2) the heterozygote codominant model (AC vs. AA), and (3) the homozygote codominant model (CC vs. AA) model [11]. For rs16886165 polymorphism, pooled OR was obtained by an allele contrast model (G vs. T). The *I^2^*-test was performed to assess possible heterogeneity among studies [12]. Hardy-Weinberg equilibrium (HWE) was tested by the *x^2^* test. Pooled odds ratios (ORs) with 95% confidence intervals (95% CIs) were calculated using fixed-effects (Mantel-Haenszel method) [13] or random-effects (DerSimonian-Laird method) [14] models. When the effects were assumed to be homogeneous, the fixed-effects model was then used; otherwise, the random-effects model was more appropriate. Subgroup analyses were performed by ethnicity, sample size (<1000 cases and >1000 cases) and estrogen receptor (ER) status (ER-positive and ER-negative). Sensitivity analyses were performed to evaluate the stability of the results by sequential omission of individual studies in the meta-analysis to show the influence of the individual data set to the pooled OR. The Egger's test and funnel plots were utilized to provide diagnosis of publication bias [15]. All of the above analyses were performed using STATA version 12.0 (StataCorp, College Station, TX).

For each statistically significant association, the false-positive report probability (FPRP) analysis was performed using the method reported by Wacholder et al. [16]. A prior probability of 0.001 was set to detect an OR of 1.20. An FPRP cutoff value of 0.2 was used, and only the results with FPRP values less than 0.2 were referred as noteworthy. The Excel spreadsheet provided by Wacholder et al. was used to calculate statistical power and FPRP values (http://jnci.oxfordjournals.org/content/96/6/434/suppl/DC1).

## Results

### Study Characteristics

**Figure 1:**
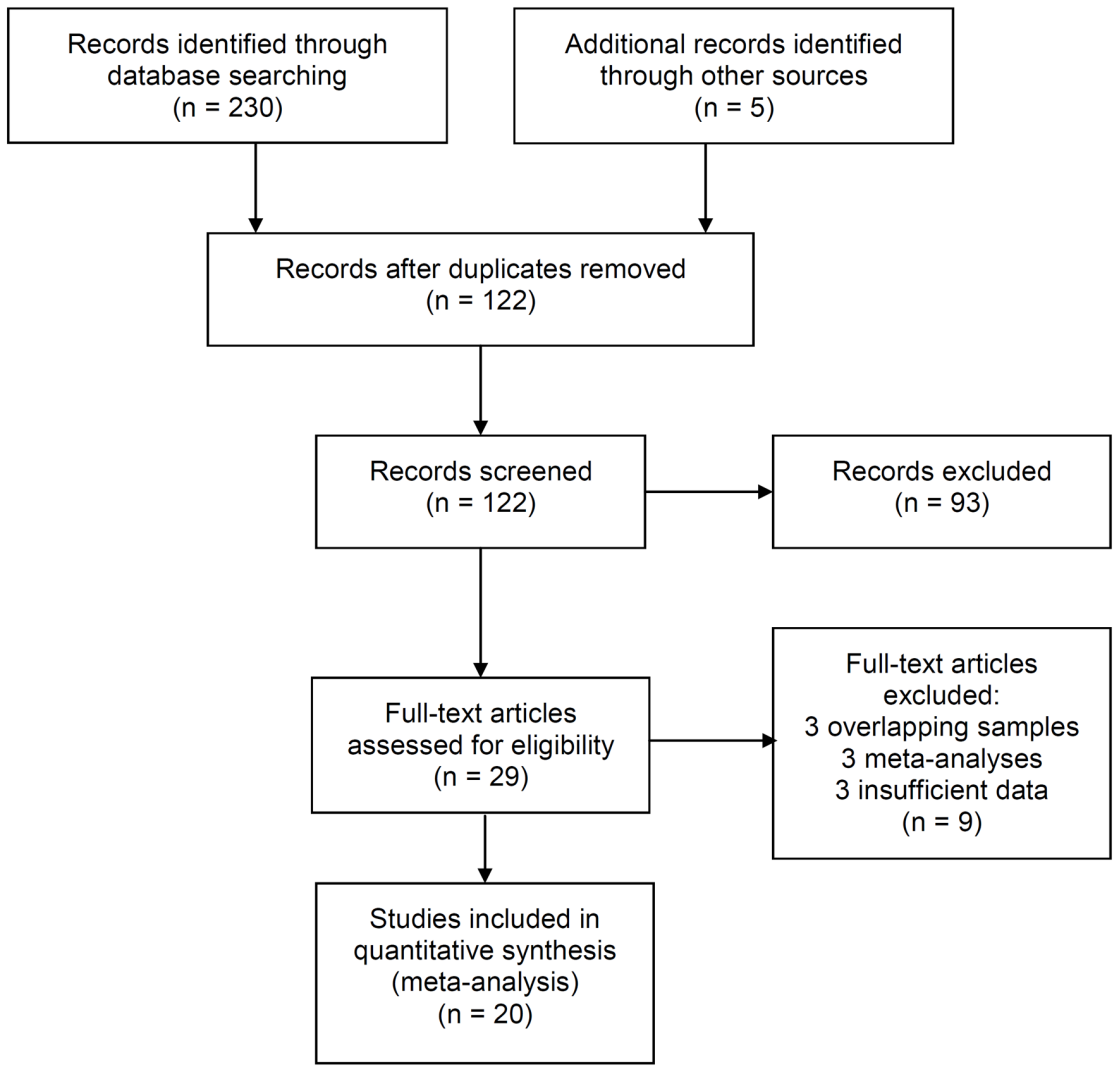
Flowchart of the study selection process.

Based on the above criteria, a total of 20 eligible studies involving 59,670 cases and 66,862 controls met all selection criteria for the pooled analyses [6], [8], [9], [17]–[33]. Figure 1 illustrates the study selection process and Table 1 describes the main characteristics of these studies. For the rs889312 polymorphism, 18 studies including a total of 54674 cases and 64542 controls were included in the meta-analysis. For the rs16886165 polymorphism, 4 studies involved a total of 7949 cases and 7660 controls. Of the cases, 67% were Europeans, 21% were Asians, and 10% were of African descendants. The extended-quality scores ranged from 5 to 8, and 19 were given high quality, while the remaining 1 study was given median quality. The distributions of genotypes in the controls in 18 studies were consistent with the Hardy-Weinberg equilibrium, but those in 2 other studies remained unknown.

**Table 1:** Characteristics of studies included in this meta-analysis.

Reference	Year	Country	Ethnicity	Polymorphism	Cases	Controls	Control source	Genotyping method	Age	Quality score	HWE of controls	Matching criteria
Garcia-Closas [6]	2008	Australia, Europe, Thailand, US	European, Asian	rs889312	21347	26081	PB, HB, Nested	iPLEX, Taqman	17–96	High	Yes	Ethnicity
Rebbeck [17]	2009	US	European, African	rs889312	685	1124	PB	SNaPshot	50–79	High	Yes	Ethnicity and age
Barnholtz-Sloan [18]	2010	US	European, African	rs889312	1971	1776	PB	GoldenGate	20–74	High	Yes	Ethnicity and age
Gorodnova [19]	2010	Russia	European	rs889312	140	174	PB	Real-time PCR	28–78	Medium	Yes	Region
Latif [20]	2010	UK	European	rs889312	870	365	HB	Taqman	18–81	High	Yes	Ethnicity
Tamimi [21]	2010	Sweden	European	rs889312	680	737	PB	iPLEX, Taqman	50–74	High	Yes	Age
Zheng [22]	2010	China	Asian	rs889312	3039	3082	PB	iPLEX, Taqman	25–64	High	NA	Age
Campa [23]	2011	US, Europe	European, African, Asian	rs889312	8576	11892	Nested	Taqman	62	High	Yes	Ethnicity and age
Chen [24]	2011	US	African	rs889312, rs16886165	3016	2745	PB, HB, Nested	BeadChip	22–87	High	NA	NA
Han [25]	2011	Korea	Asian	rs889312	3296	3497	PB	Taqman	21–82	High	Yes	Region
Jiang [8]	2011	China	Asian	rs889312	493	510	PB	SNaPshot	49.5	High	Yes	Ethnicity and age
Slattery [26]	2011	US	European	rs889312	1735	2041	PB	TaqMan	25–79	High	Yes	Ethnicity and age
Butt [27]	2012	Sweden	European	rs889312	689	1385	PB	iPLEX	NA	High	Yes	Age and time of sampling at baseline
Chan [9]	2012	China	Asian	rs889312, rs16886165	1174	1485	PB	Taqman	51	High	Yes	NA
Harlid [28]	2012	Europe	European	rs889312	3554	5015	PB	iPLEX	22–95	High	Yes	Age and time of sampling at baseline
Kim [29]	2012	Korea	Asian	rs889312, rs16886165	2257	2052	PB	TaqMan	NA	High	Yes	NA
Shan [30]	2012	Arab	European	rs889312	640	371	PB	TaqMan	23–91	High	Yes	Age
Jara [31]	2013	chili	NA	rs889312	469	351	HB	TaqMan	42.3	High	Yes	Age and socioeconomic strata
Resler [32]	2013	US	NA	rs889312	840	801	PB	GoldenGate	65–79	High	Yes	Age
Zheng [33]	2013	US	African	rs16886165	1502	1378	PB, HB	GoldenGate	48.1	High	Yes	NA

NA: not available; PB, Population-based; HB, Hospital-based; Nested, nested case-control study; HWE, Hardy-Weinberg equilibrium.

### Meta-analysis results

**Figure 2:**
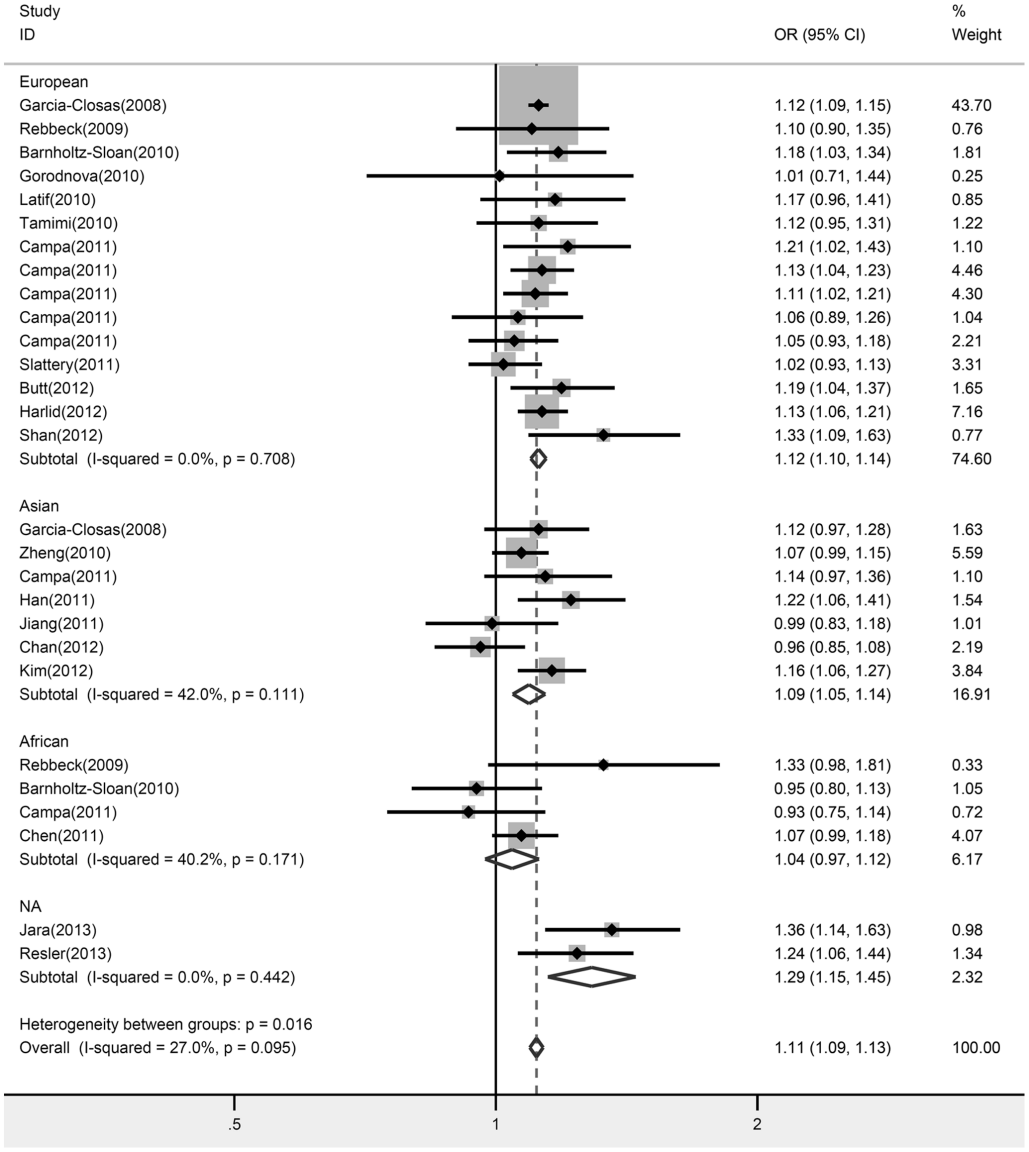
Forest plot of *MAP3K1* rs889312 polymorphism and breast cancer risk stratified by ethnicity. Fixed-effect model was used for the analysis (allele contrast model C vs. A).

**Figure 3:**
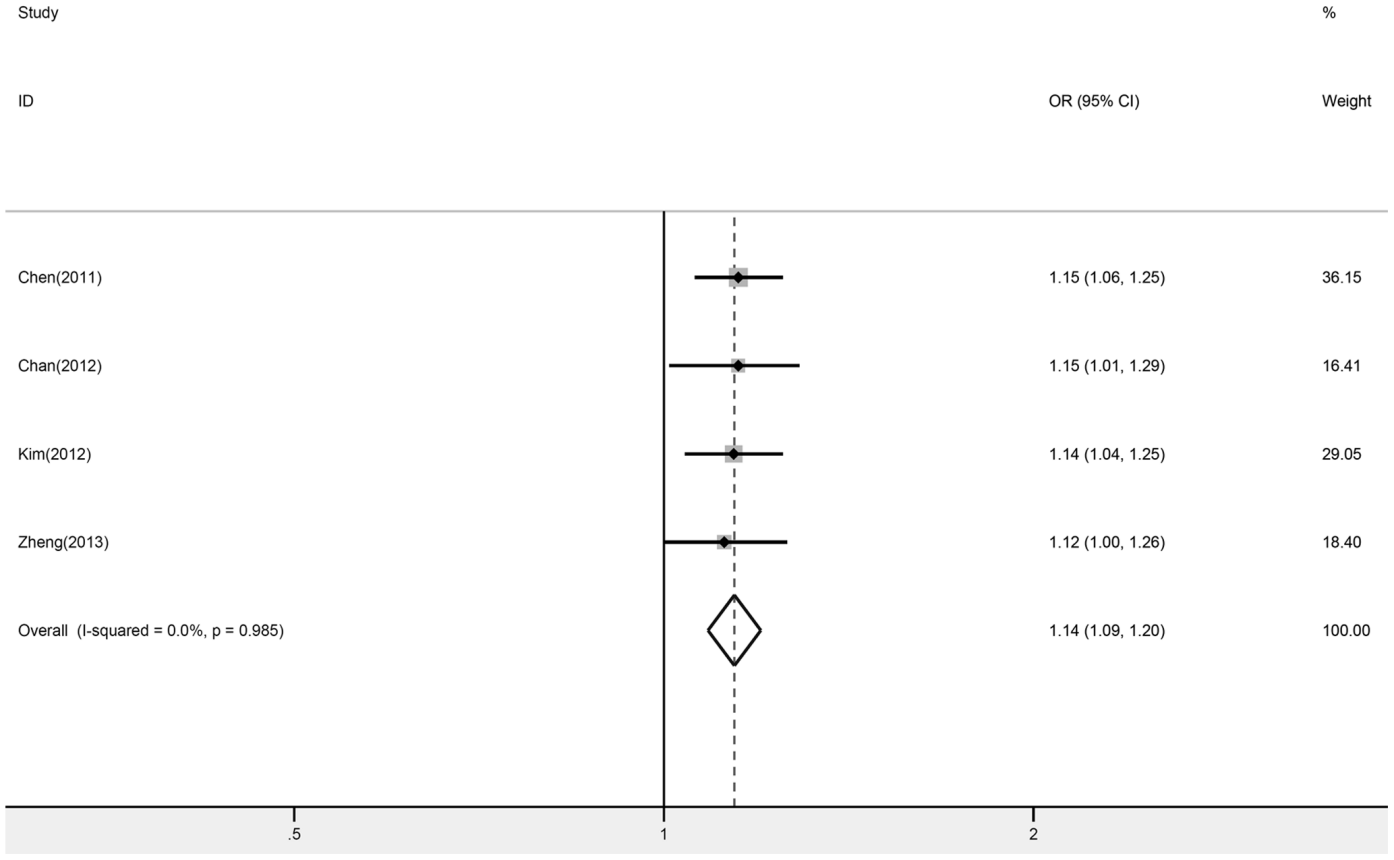
Forest plot of *MAP3K1* rs16886165 polymorphism and breast cancer risk. Fixed-effect model was used for the analysis (allele contrast model G vs. T).

Overall, the rs889312 polymorphism was significantly associated with increased breast cancer risk under all genetic models (C vs. A: OR = 1.11, 95% CI: 1.09–1.13, *P* = 0.000, I^2^ = 27.0%; AC vs. AA: OR = 1.09, 95% CI: 1.05–1.13, *P* = 0.000, I^2^ = 20.0%; CC vs. AA: OR = 1.21, 95% CI: 1.15–1.28, *P* = 0.000, I^2^ = 31.9%; Figure 2). Additionally, the meta-analysis resulted in a statistically significant association between rs16886165 and breast cancer. The pooled OR for risk G allele was 1.14 under allele contrast model (95% CI: 1.09–1.20, *P* = 0.000, I^2^ = 0.0%; Figure 3).

**Figure 4:**
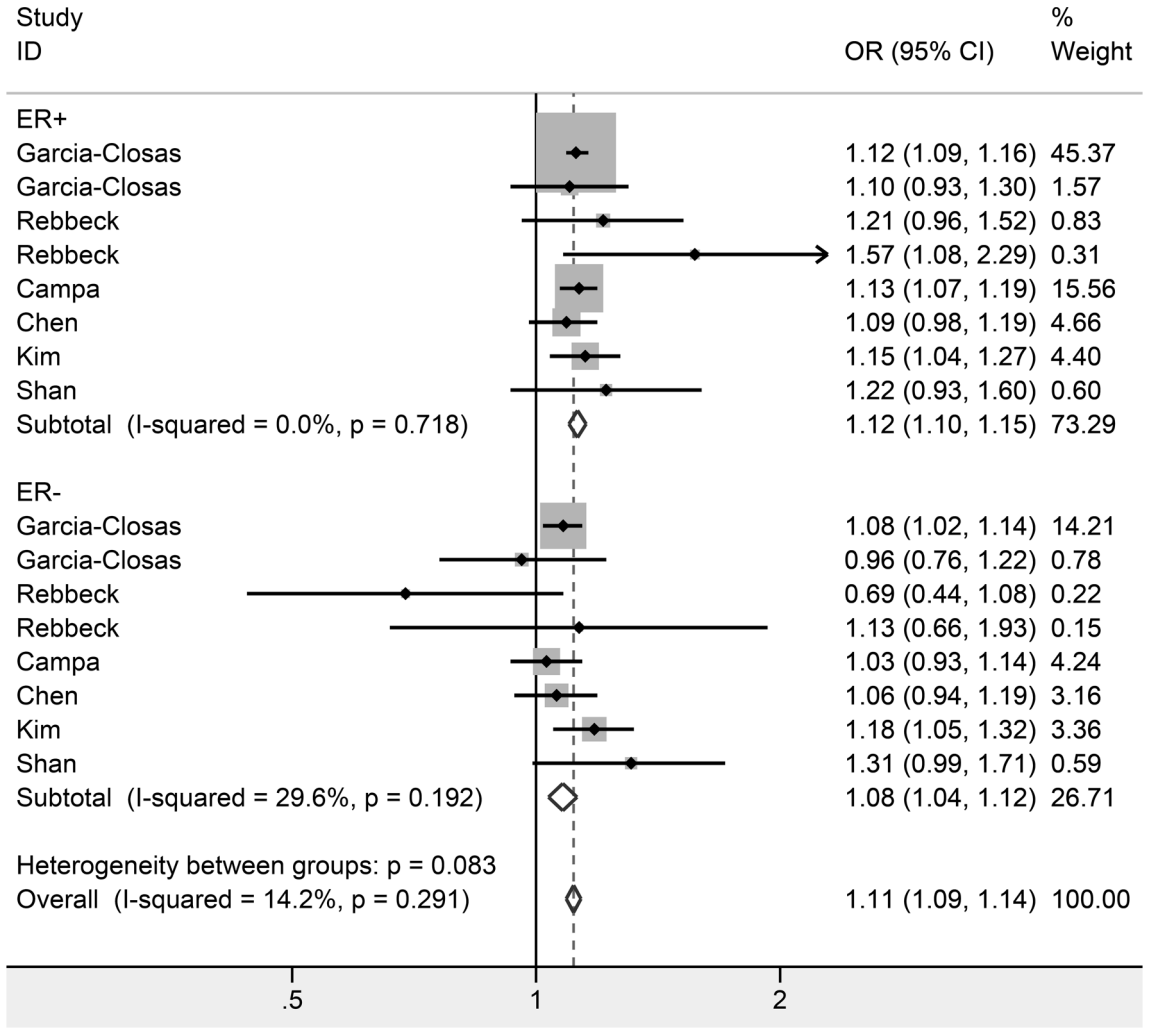
Forest plot of *MAP3K1* rs889312 polymorphism and breast cancer risk stratified by ER status. Fixed-effect model was used for the analysis (allele contrast model C vs. A).

In the subgroup analysis by ethnicity, our results indicated a significant association between the SNP rs889312 and breast cancer incidence in European ancestry populations (C vs. A: OR = 1.12, 95% CI: 1.10–1.14, *P* = 0.000, I^2^ = 0.0%; AC vs. AA: OR = 1.12, 95% CI: 1.08–1.18, *P* = 0.000, I^2^ = 0.0%; CC vs. AA: OR = 1.24, 95% CI: 1.15–1.33, *P* = 0.000, I^2^ = 0.0%). For Asians, two genetic comparisons produced significantly increased risks (C vs. A: OR = 1.09, 95% CI: 1.05–1.14, *P* = 0.000, I^2^ = 42.0%; CC vs. AA: OR = 1.15, 95% CI: 1.05–1.26, *P* = 0.002, I^2^ = 17.8%), but the heterozygote codominant model did not reach statistically significance. Among Africans, we did not detect any significant association under all genetic models (Table 2). According to sample size, the rs889312 polymorphism presented significantly increased risks of breast cancer both in small and large studies. The data on rs889312 polymorphism stratified by ER status were available in 6 studies involving 29,200 cases and 44,104 controls. Subsidiary analyses of ER status yielded a per-allele OR for ER-positive tumors of 1.12 (95% CI: 1.10–1.15, *P* = 0.000, I^2^ = 0.0%) and ER-negative tumors of 1.08 (95% CI: 1.04–1.12, *P* = 0.000, I^2^  =  29.6%; Figure 4). FPRP values at the pre-specified prior probability of 0.001 were lesser than 0.2, except associations among Asians under the homozygote codominant model and small group under the heterozygote codominant model (Table 2).

**Table 2:** Stratified analyses for *MAP3K1* rs889312 polymorphism and breast cancer risk.

Analyses	Allele contrast model					Heterozygote codominant model					Homozygote codominant model				
	OR (95% CI)	P(Z)	I^2^	Power OR,1.2	FPRP	OR (95% CI)	P(Z)	I^2^	Power OR,1.2	FPRP	OR (95% CI)	P(Z)	I^2^	Power OR,1.2	FPRP
Overall	1.11(1.09–1.13)	0.000	27.0%	1.000	0.000	1.09(1.05–1.13)	0.000	20.0%	1.000	0.003	1.21(1.15–1.28)	0.000	31.9%	0.386	0.000
Ethnicity															
European	1.12(1.10–1.14)	0.000	0.0%	1.000	0.000	1.12(1.08–1.18)	0.000	0.0%	0.995	0.020	1.24(1.15–1.33)	0.000	0.0%	0.180	0.000
Asian	1.09(1.05–1.14)	0.000	42.0%	1.000	0.142	1.02(0.95–1.11)	0.560	37.2%	1.000	0.998	1.15(1.05–1.26)	0.002	17.8%	0.819	0.768
African	1.04(0.97–1.12)	0.239	40.2%	1.000	0.997	0.95(0.80–1.12)	0.544	39.3%	0.941	0.998	1.03(0.78–1.36)	0.820	51.3%	0.859	0.999
NA	1.29(1.15–1.45)	0.000	0.0%	0.113	1.148	1.14(0.96–1.34)	0.134	0.0%	0.733	0.993	1.86(1.42–2.43)	0.000	0.0%	0.001	0.891
Sample size															
<1000	1.12(1.07–1.16)	0.000	35.2%	1.000	0.000	1.09(1.02–1.17)	0.011	27.7%	0.996	0.945	1.32(1.19–1.47)	0.000	27.5%	0.041	0.010
>1000	1.11(1.09–1.14)	0.000	18.8%	1.000	0.000	1.09(1.05–1.14)	0.000	14.7%	1.000	0.142	1.18(1.11–1.26)	0.000	29.3%	0.692	0.001

### Sensitivity analyses and publication bias

**Figure 5:**
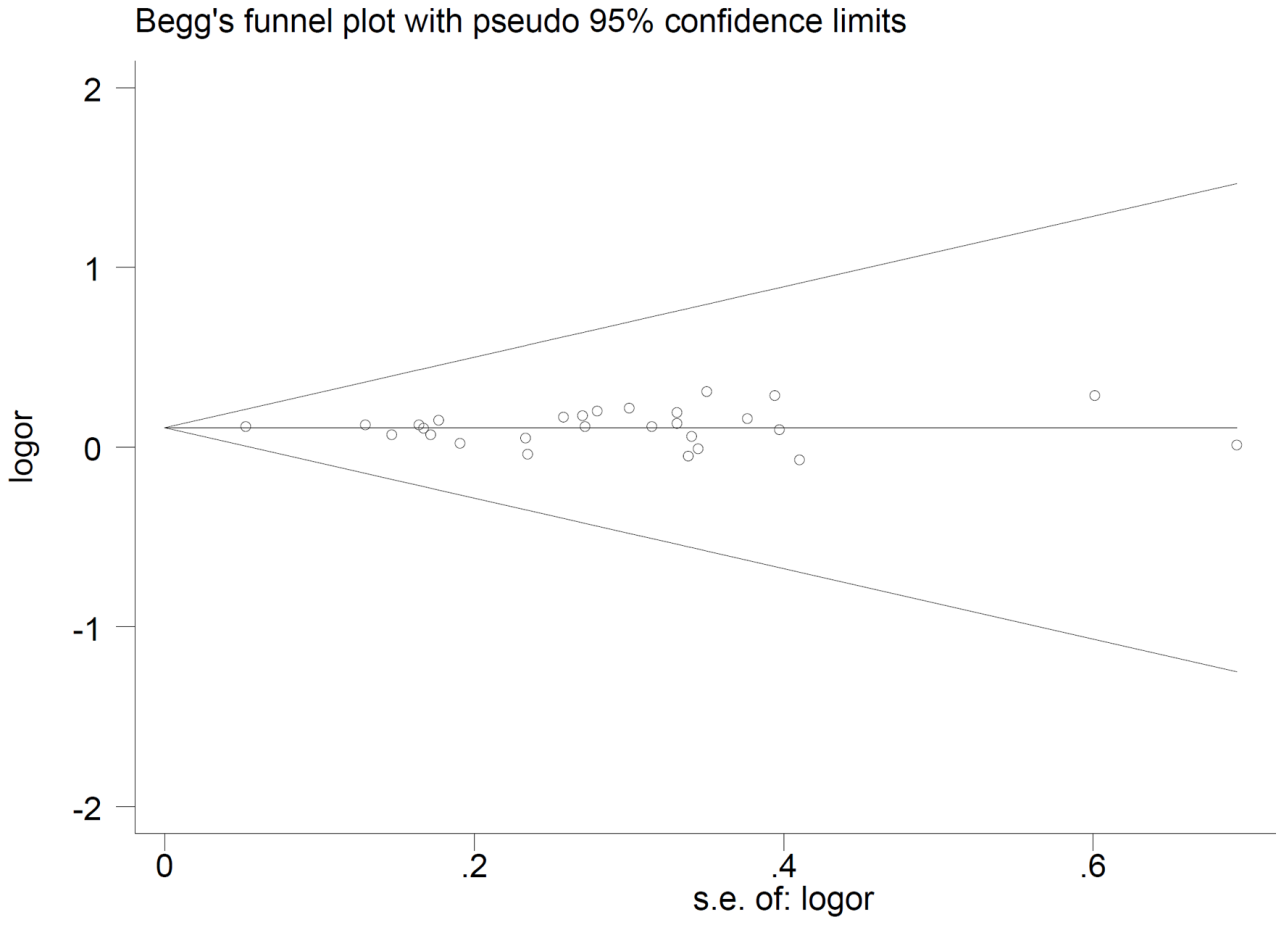
Begg’s funnel plot of *MAP3K1* rs889312 polymorphism and breast cancer risk.

**Figure 6:**
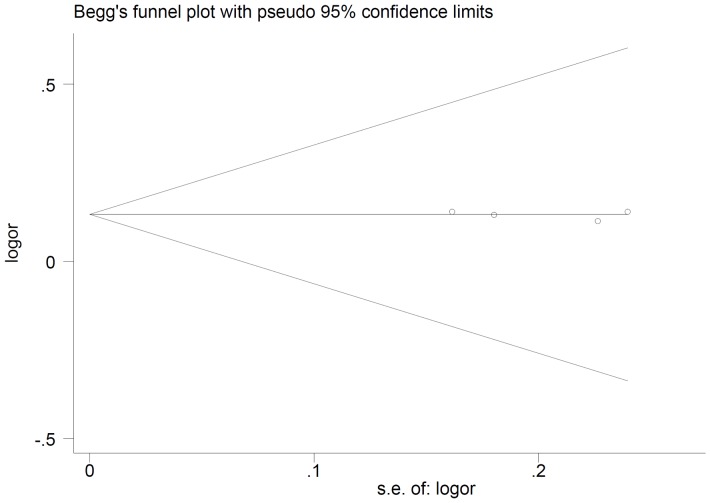
Begg's funnel plot of *MAP3K1* rs16886165 polymorphism and breast cancer risk.

A sensitivity analysis was performed by sequential removal of individual studies. The results suggested that no individual study significantly altered the pooled ORs. The publication bias of the studies was evaluated by funnel plot and Egger's test. As shown in Figure 5 and 6, the shape of the funnel plots did not reveal obvious evidence of asymmetry for both polymorphisms. All the p values of Egger's tests were more than 0.05, suggesting that no publication bias was detected in these studies.

## Discussion

The common variants rs889312 and rs16886165 lie in a linkage disequilibrium (LD) block of approximately 280 kb which includes *MAP3K1* gene [31]. The *MAP3K1* gene, which is also known as *MEKK1* (Mek kinase 1), encodes a 196-kDa serine/threonine protein kinase that activates the ERK (extracellular signal-regulated kinase), JNK (c-Jun NH2-terminal kinase) and NF-κB (nuclear factor-κB) pathways [34]. The downstream signal transductions regulate the survival, differentiation, proliferation and apoptosis of cell, and appear to be involved in tumor development and tumor progression [35]–[37]. As rs16886165 and rs889312 are 88.3 and 79.5 kb upstream of MAP3K1 respectively, the causal variant may be closer to rs16886165 than rs889312 [9]. Although these two SNPs do not change the structure and therefore the biochemical function of MAP3K1, they may have effects on the modulation of MAP3K1 expression and therefore the tuning of MAPK signal transductions.

This meta-analysis demonstrated that the rs889312 and rs16886165 SNPs in *MAP3K1* were associated with increased breast cancer susceptibility. There are a number of meta-analyses that have been performed to elucidate the associations between SNPs and breast cancer risk in recent years; however, only 21.7% of those significant associations were noteworthy [38]. Therefore, we performed FPRP analyses in this work to confirm robustness of the linkages. Although a previous meta-analysis directed by Lu et al. [11] has demonstrated that the rs889312 polymorphism was significantly correlated with breast cancer risk, it only included seven case-control studies without subgroup analysis. In the current meta-analysis, we have made much more powerful and detailed analysis to support our results: (1) more studies were included; (2) subgroup analysis was conducted and stratified by ethnicity and sample size; (3) the association between ER status and breast cancer was considered.

When stratified by ethnicity, the rs889312-C allele showed to be a risk factor for the development of breast cancer in European and Asian ancestry populations, but not in Africans. Different frequencies of mutant alleles in different ethnic groups may contribute to different susceptibilities to cancer [39]. Different life styles and environmental factors among the different populations lead to diverse gene-environment interactions, and may therefore also account for different cancer susceptibilities [40]. In addition, the result may be partly biased because of the limited number of studies in Africans, which had insufficient power to detect a different linkage disequilibrium (LD) pattern or minor effect of the SNP among African populations. Large population studies, which significantly reduce the evidence from smaller studies, play dominant roles in the meta-analysis. When considering sample size, we found rs889312 was associated with increased breast cancer risk both in large and small studies under all genetic models. For rs16886165, the available studies were insufficient to stratified analysis. The pooled result of the current meta-analysis indicated that the rs16886165-G allele conferred breast cancer risk in Asian and African ancestry population. Additionally, rs16886165 have been identified as a low-penetrance risk factor for breast cancer in European ancestry population by GWAS [7].

The prognosis of breast cancer is affected by ER status. Previous studies suggested that rs889312 was related to ER-positive breast cancers, which was confirmed by our stratified analysis by ER status. For ER-negative tumors, the results of previous studies were contradictory, but this meta-analysis showed rs889312 might also confer risk. Furthermore, the frequencies of the rs889312-C allele in *MAP3K1* are similar in ER-positive and ER-negative tumors.

Despite the strengths of this study, such as the large sample size, no significant heterogeneity and high quality of the qualified studies, there are several limitations that should be addressed. First, the sample size of Africans was relatively small, thus the analyses might have insufficient statistical power to detect an association. The trial sequential analysis (TSA) can be used to reveal insufficient information size and potentially false positive results in meta-analyses [41]. However, we are unable to do TSA due to the lack of detailed information which is needed for TSA. Future larger sample studies are necessary to clarify more exact associations between these SNPs in *MAP3K1* and breast cancer in African descendants. Second, we performed the analyses under heterozygote codominant model, homozygote codominant model and the subgroup analysis by ER status on a fraction of all data, the selection bias might therefore have occurred. Third, the controls were not uniformly defined, for those based on hospital population might have had benign disease and had different risks for developing breast cancer. Therefore, non-differential misclassification bias might be possible. Finally, due to the lack of individual-level data, the results were based on unadjusted published estimates. We were unable to examine the interactions of possible confounders including age, menopausal status, obesity, smoking, alcohol consumption and environmental factors.

In summary, this meta-analysis indicated that the polymorphism rs889312 was associated with breast cancer risk in Europeans and Asians, while rs16886165 was a risk factor for breast cancer in Asian and African women, and both could be served as markers predisposition to breast cancer. Larger sample studies by using homogeneous patients, unbiased genotyping methods, as well as well-matched controls will provide further supporting information on association of *MAP3K1* polymorphisms rs889312 and rs16886165 in breast cancer predisposition.

## Supporting Information

Checklist S1 PRISMA checklist for this meta-analysis.
(DOC)
